# Enhanced Autophagy of Adipose-Derived Stem Cells Grown on Chitosan Substrates

**DOI:** 10.1089/biores.2014.0032

**Published:** 2015-01-01

**Authors:** Ching-Ming Yang, Yen-Jang Huang, Shan-hui Hsu

**Affiliations:** ^1^ Institute of Polymer Science and Engineering, National Taiwan University, Taipei, Taiwan.; ^2^ Research Center for Developmental Biology and Regenerative Medicine, National Taiwan University, Taipei, Taiwan.

**Keywords:** autophagy, mesenchymal stem cells, oxidative stress, spheroid

## Abstract

Autophagy is an important protein quality control mechanism for cells under stress conditions to promote cell survival. Modulation of autophagy on biomaterial substrates is rarely reported. In this study, the autophagy of adipose-derived stem cells (ADSCs) cultured on chitosan (CS) substrates was examined. Compared to the traditional monolayer culture, ADSCs cultured on CS substrates showed spheroid formation as well as a prolonged upregulation of autophagosomal marker-microtubule-associated protein 1 light chain 3 (LC3) II protein expression. In addition, the green fluorescent protein tagged-LC3 (GFP-LC3) expressing ADSCs also revealed more GFP-LC3 puncta on CS substrates. The enhanced autophagy on CS substrates was associated with Ca^2+^, while ethylene glycol tetraacetic acid (EGTA), a Ca^2+^ chelator, repressed the autophagy in a dose-dependent manner. Moreover, ADSC spheroids on CS substrates demonstrated a higher survival rate and autophagy response upon H_2_O_2_ treatment. The upstream components of autophagy signal pathway-UNC51-like kinase 1 (*Ulk1*), autophagy-related protein 13 (*Atg13*), and autophagy/beclin-1 regulator 1 (*Ambra1*) genes were more highly expressed in ADSC spheroids before and after adding H_2_O_2_ than those in the conventional culture. EGTA also decreased the cell viability and autophagy-associated gene expression for ADSC spheroids on CS substrates after H_2_O_2_ treatment. Therefore, we suggest that three-dimensional (3D) cell culture on CS may confer ADSCs the ability to increase the autophagic flux in response to stimulations in a Ca^2+^-dependent manner.

## Introduction

Autophagy is a conserved catabolic mechanism for degradation of nonessential or dysfunctional cellular organelles and proteins.^1,2^ Upon induction, the Ulk1 protein kinase complex (Ulk1, Atg13, FIP200, and Atg101) initiates autophagosome formation^3^ and the autophagy/beclin-1 regulator 1 (Ambra1) enhances Ulk1 kinase activity.^4^ Microtubule- associated protein 1 light chain 3-II (LC3-II) localized in the double-membranes of autophagosome is considered a proper marker to monitor autophagy.^5^ Besides, cytosolic Ca^2+^ is important for autophagy induction.^6,7^

Mesenchymal stem cells (MSCs) from different adult tissues have recently become a potential cell source for cell therapy because they can differentiate into a variety of cell types.^8^ The environmental conditions of host tissues, however, may not be suitable for MSC growth (e.g., inflammation, hypoxia, oxidative stress, or lack of nutrients). The poor cell viability may further lead to the limited therapeutic efficacy.^9^ Recent works have shown that autophagy protects the bone marrow-derived MSCs from apoptosis under hypoxia and serum deprivation.^10–12^

Chitosan (CS) is a polymer obtained from deacetylation of chitin, a nitrogen-containing polysaccharide abundant in nature. The thin membranes made of CS can facilitate cell–cell interaction and induce the self-assembly of MSCs to form three-dimensional (3D) spheroids on the membranes.^13^ These 3D spheroids express a greater level of stemness marker genes (*Oct4*, *Sox2*, and *Nanog*) and have higher differentiation and engrafting potentials.^14,15^ CS-derived MSC spheroids showed higher cell survival rates in an inflammatory milieu of a sciatic nerve gap^16^ or an infarcted myocardium in rats.^17^ The enhanced cell survival may suggest a protection mechanism from apoptosis.

The purpose of this study is to investigate the regulation of autophagy in adipose-derived stem cells (ADSCs) grown on CS substrates. Here, we demonstrated that culturing on CS substrates increased autophagy in ADSCs and this process required Ca^2+^. Finally, we suggested that enhanced autophagy may contribute to the cell survival under oxidative stress and nutrient starvation in CS-derived ADSC spheroids.

## Materials and Methods

### Cell culture and transfection

All procedures followed the ethics guidelines and were approved by the Animal Care and Use Committee of the University. ADSCs were isolated from the adipose tissue of Sprague-Dawley rats (body weight from 250 to 500 g).^13^ ADSCs were cultured in Dulbecco's modified Eagle's medium–low glucose/F12 (1:1) supplemented with 10% fetal bovine serum (FBS), 100 μg/ml streptomycin, and 100 U/ml penicillin (all from Gibco) at 37°C in a 5% CO_2_ atmosphere. Cells of the third to the fifth passages were used in this study. Rat LC3 cDNA was inserted to pEGFP-C1 vector (Clontech) to create a green fluorescent protein tagged-LC3 protein expressing vector.^5^ ADSCs were seeded in a 6-well culture plate for 24 h before transfection. Two micrograms of GFP-LC3 plasmids was transfected into ADSCs using the K2^®^ Transfection System (Biontex) according to the manufacturer's instructions.

### Preparation of CS substrates and spheroid formation

To prepare 1% CS solution, CS powder (Sigma; molecular weight 510 kDa and degree of deacetylation 77%) was dissolved and stirred in 1% aqueous acetic acid solution at room temperature for 16 h. After filtration by a 100 μm mesh, the 1% CS solution was casted on a 24-well tissue culture plate (300 μl/well) or 6-well tissue culture plate (1.5 ml/well) and air-dried in a laminar cabinet for 24 h. Then 0.5 N NaOH in 75% ethanol was added to the CS substrates for 5 min and washed with distilled water for three times. For spheroid formation, 5×10^4^ or 1×10^4^ cells were seeded in each well of the CS-coated 24- or 96-well tissue culture plate.

### H_2_O_2_ treatment and WST-1 assay

ADSCs (1×10^4^) were seeded in the blank culture well (tissue culture polystyrene, TCPS) or CS-coated 96-well culture plate. Cells were cultured for 24 h on TCPS or 72 h on CS substrates. The medium was replaced in two groups for ADSCs grown on TCPS: a control group, and a H_2_O_2_-treated group (incubation with 600 μM H_2_O_2_ in 2% FBS medium). The medium was replaced in four groups for ADSCs grown on CS substrates: a control group, a H_2_O_2_ treated-group (incubation with 600 μM H_2_O_2_ in 2% FBS medium), a H_2_O_2_+ethylene glycol tetraacetic acid (EGTA)-treated group (incubation with 600 μM H_2_O_2_ and 2 mM EGTA in 2% FBS medium); and an EGTA-treated group (incubation with 2 mM EGTA in 2% FBS medium). After 2 h incubation, cell viability was detected by the cell proliferation reagent WST-1 kit (Roche Applied Science) according to the manufacturer's instructions.

### Western blots analysis

ADSCs were seeded on TCPS or CS substrates at a density of 2.5×10^5^ cells per well in a 6-well culture plate and cultured for 0, 2, 4, 8, 16, 24, and 48 h. ADSCs were harvested at the indicated times. For H_2_O_2_ treatment, ADSCs were cultured for 24 h on TCPS or 72 h on CS substrates. After 2 h incubation, ADSCs were harvested. Cells were lysed in lysis buffer containing 20 mM Hepes (pH 7.5), 420 mM NaCl, 1.5 mM MgCl2, 0.1% NP-40, and protease inhibitor cocktail (Sigma). Proteins were separated by 15% SDS-PAGE and the endogenous LC3 proteins were detected by rabbit polyclonal anti-LC3 antibody (Genetex). For internal control, GAPDH proteins were detected by rabbit monoclonal anti-GAPDH antibody (Cell Signaling). The intensities of protein bands were quantified by Labwork software (UVP).

### RNA extraction and quantitative RT-PCR analysis

ADSCs were seeded in the TCPS or CS substrates at a density of 5×10^4^ cells per well in a 24-well culture plate and were cultured 24 h for TCPS or 72 h for CS substrates. After 2 h incubation with H_2_O_2_, total RNAs were isolated with Trizol reagent (Invitrogen) and cDNA was synthesized using the RevertAid First Strand cDNA Synthesis Kit (MBI Fermentas) according to the manufacturer's instruction. RT-PCR was performed with DyNAmo Flash SYBR Green qPCR Kit (Finnzymes) on a Chromo 4 PTC200 Thermal Cycler (MJ Research). The primers used were *Ulk1* forward 5′- TGGAGAACCTAGCCAGCAGT-3′ and reverse 5′-GTGCTTCACACACGACGACT-3′; *Atg13* forward 5′-AGACCAAGCAAGTCGAAGGA-3′ and reverse 5′- CCTTTGGGAGATGATGGCTA-3′; *Ambra1* forward 5′-GCAGCTTTCATCCCGAGTAG-3′ and reverse 5′-AAGACCTGGGCTACCATGTG-3′; and *GAPDH* forward 5′- AGGTCGGTGTGAACGGATTTG-3′ and reverse 5′-GGGGTCGTTGATGGCAACA-3′. The relative quantification in gene expression was determined using the 2-ΔΔ-CT method.^18^ Using this method, the fold of changes in gene expression normalized to the *GAPDH* gene was obtained relative to TCPS.

### Immunocytochemistry

After transfection with GFP-LC3 plasmids for 24 h, ADSCs were recovered for another 24 h before seeding on CS substrates. After 24 h the spheroids were then carefully collected and washed with PBS. The spheroids were trypsinized into single cells and seeded on a 1% gelatin-coated cover slide for 4 h. After washing with PBS and fixation in 4% paraformaldehyde at room temperature for 10 min, and two washes with 1% Tween/PBS, the slide was mounted by 90% glycerol (Merk). Puncta of GFP-LC3 were analyzed using a fluorescence microscope.

### Statistical analysis

Numerical values were expressed as the mean±standard deviation (SD). Differences between two groups were assessed by unpaired two-tailed *t*-tests. Results involving more than two groups were assessed by one-way ANOVA and Tukey's multiple comparison test. A *p*-value of less than 0.05 was considered to be statistically significant. Independent experiments were performed for each type of experiments.

## Results

### Induction of autophagy in ADSCs on CS substrates

**FIG. 1.**
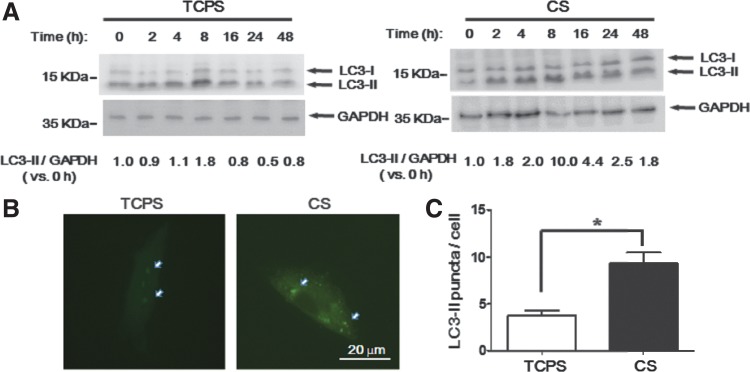
Upregulation of autophagy in ADSC spheroids. **(A)** ADSCs were seeded on CS substrates in a 24-well plate or a blank well (TCPS) and harvested at 0, 2, 4, 8, 16, 24, and 48 h. The LC3 protein expression was detected by Western blots at the indicated time points. GAPDH was used as an internal control. Band intensities were quantified, and the LC3-II expression was normalized to GAPDH. **(B)** GFP-LC3 plasmids were transfected to ADSCs and cells were seeded to CS substrates for 24 h. Cells were then transferred to a 1% gelatin-coated cover slide and fixed. The GFP-LC3 puncta were analyzed with 100×magnification using a fluorescence microscope. **(C)** Quantitative analyses of GFP-LC3 puncta per cell. *p<0.05 versus TCPS. ADSCs, adipose-derived stem cells; CS, chitosan; TCPS, tissue culture polystyrene.

To examine whether cell culture on CS substrates increased autophagy, the expression of endogenous LC3-II, an autophagosomal marker, was detected by Western blots. ADSCs on TCPS or CS substrates were analyzed at different time points. When grown on CS substrates, the LC3-II protein expression increased from 2 h and reached a high level at 8 h. Although the LC3-II protein expression decreased from 16 to 48 h, it was still higher than the starting point (Fig. 1A, right panel). The LC3-II protein expression on TCPS from 4 to 8 h also slightly increased, but the fold of increase was much lower than that on CS substrates (Fig. 1A, left panel). The formation of LC3 puncta was further examined at 24 h. Formation of GFP-LC3 puncta in ADSCs on CS substrates was clearly observed and the number of GFP-LC3 puncta was more than that on TCPS (Fig. 1B,C). These results indicated that autophagy was more pronounced and prolonged in ADSCs when cultured on CS substrates.

### EGTA inhibited autophagy of ADSCs on CS substrates

**FIG. 2.**
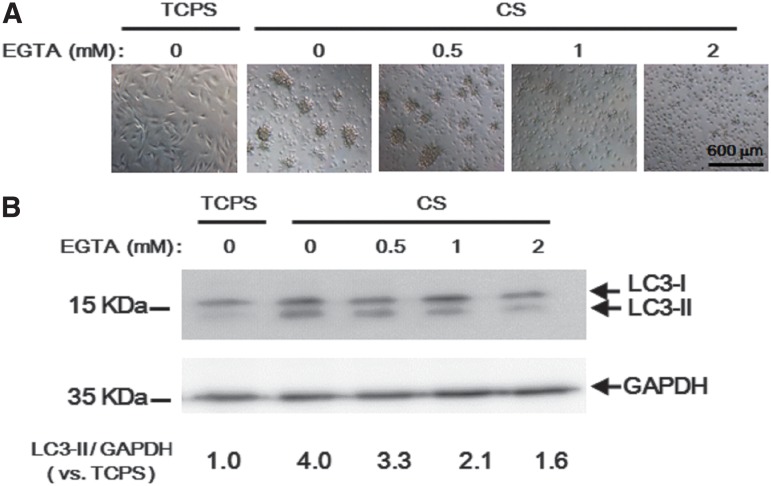
Autophagy inhibition by EGTA. **(A)** ADSCs were seeded on CS substrates in a 24-well plate with the medium containing 0, 0.5, 1, or 2 mM EGTA or in a blank well (TCPS) for 8 h. The morphologies of ADSCs were taken by the microscope. **(B)** Cell lysates were collected after treatment and subjected to Western blot analysis with anti-LC3 for LC3-II. GAPDH was used as an internal control. Band intensities were quantified and normalized to GAPDH. EGTA, ethylene glycol tetraacetic acid.

Since the increase in cytosolic Ca^2+^ could induce autophagy,^6,7^ we sought to investigate whether Ca^2+^ chelation affected the autophagy of ADSCs on CS substrates. To this end, ADSCs were cultured in the medium containing 0.5–2 mM EGTA (a Ca^2+^ chelator). After 8 h, the morphology of ADSCs and LC3-II protein expression level were examined. It was observed that 0.5 mM EGTA had no effect on sphere formation, but for concentrations greater than 1–2 mM EGTA the sphere formation was obviously inhibited (Fig. 2A). Western blots showed that when the EGTA concentration increased, the LC3-II protein expression gradually decreased for ADSCs on CS substrates (Fig. 2B). On CS, EGTA at 0.5 mM slightly decreased the LC3-II protein expression of ADSCs. The LC3-II protein expression of ADSCs on CS in the presence of 2 mM EGTA was similar to that on TCPS without EGTA. These results suggested that Ca^2+^ may be required for the upregulation of ADSC autophagy on CS substrates.

### Enhanced cell survival of ADSCs on CS substrates under oxidative stress may be related to autophagy

**FIG. 3.**
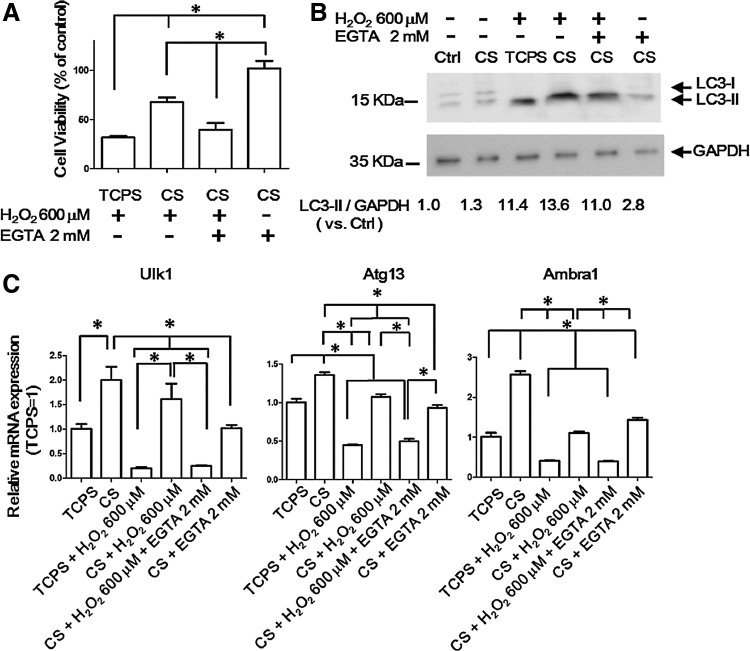
Autophagy-protecting ADSCs against oxidative stress and low-nutrient condition. **(A)** Cell viability was estimated by WST-1 assay after 600 μM H_2_O_2_ or 2 mM EGTA treatment in 2% FBS medium for 2 h. *p<0.05. **(B)** ADSCs were cultured on TCPS for 24 h or CS 72 h. After treatment as described in **(A)** for 2 h, cells were harvested and subjected for Western blots with anti-LC3 antibodies. ADSCs grown on TCPS for 24 h without treatment were defined as the control group (ctrl). Band intensities were quantified and normalized to *GAPDH* (the internal control). **(C)** *ULk1*, *Atg13*, and *Ambra1* mRNA levels were determined by quantitative RT-PCR after treatment as described in **(A)**. *p<0.05.

We hypothesized that the enhanced autophagy in CS-derived ADSC spheroids might reduce cell apoptosis under oxidative stress. To mimic the *in vivo* condition, following cell culture on TCPS for 24 h or on CS substrates for 72 h, the cells were treated with the medium containing 2% FBS and 600 μM H_2_O_2_ for 2 h before the cell viability was assessed. H_2_O_2_ treatment significantly reduced the cell survival of ADSCs grown on TCPS. On the other hand, ADSCs grown on CS substrates showed a higher percentage of cell survival (Fig. 3A). The LC3-II protein expression was also higher for ADSCs grown on CS substrates than on TCPS under the H_2_O_2_ treatment (Fig. 3B). Furthermore, adding 2 mM EGTA with H_2_O_2_ to the ADSCs grown on CS substrates decreased both the cell viability and LC3-II protein expression (Fig. 3A,B). EGTA treatment without H_2_O_2_ for 2 h presented minor effects to cell viability and LC3-II protein expression for ADSCs grown on CS substrates (Fig. 3A,B). These results implied that the autophagy of CS-derived ADSC spheroids increased and protected them from the oxidative stress and nutrient deprivation in a calcium-dependent manner.

To investigate the possible mechanisms behind the enhanced autophagy of CS-derived ADSC spheroids under the oxidative stress, the mRNA expression of autophagy upstream components was examined. It was noted that *Ulk1*, *Atg13*, and *Ambra1* genes were all upregulated for ADSCs grown on CS substrates (Fig. 3C). After H_2_O_2_ treatment, the expression of *Ulk1*, *Atg13*, and *Ambra1* mRNA was decreased for both ADSCs cultured on CS substrates and those cultured on TCPS. However, the mRNA expression was still much higher in CS-derived ADSC spheroids (Fig. 3C). EGTA inhibited the mRNA expression of these genes in CS-derived ADSC spheroids under H_2_O_2_ treatment. These results suggested that the enhanced autophagy in CS-derived ADSC spheroids may be associated with the upregulation of the autophagy upstream genes *Ulk1*, *Atg13*, and *Ambra1*.

## Discussion

Various *in vitro* preconditioning methods have been developed to enhance the efficacy of transplanted MSCs.^19^ Hypoxia-induced autophagy can protect MSCs against apoptosis under oxidative stress.^11,12^ MSCs with hypoxic preconditioning significantly improve cell survival after transplantation.^20^ MSC spheroids derived from CS substrates were also shown to have better differentiation capacity and engrafting potential.^16,17^ Our previous study demonstrated that CS-derived ADSC spheroids once transplanted in a chronic myocardial infarction animal model had better therapeutic effects than single cells.^17^ The ischemic area usually exhibits oxidative stress and nutrient deprivation. In this study we demonstrated that autophagy was significantly enhanced in CS-derived ADSC spheroids. Inhibition of autophagy by EGTA treatment decreased the cell viability under the oxidative stress and nutrient deprivation. These results suggested that autophagy may be an important pathway against oxidative stress to enhance the engraftment efficiency for CS-derived MSC spheroids or hypoxia-preconditioned MSCs. Moreover, spermidine and resveratrol that could activate autophagy were used to extend longevity in various model organisms.^21–23^ CS-derived MSC spheroids maintained the expression of stemness marker genes.^13^ We thus further suggested that the enhanced autophagy in ADSC spheroids may not only protect cells form stress but also prevent early senescence during cell expansion *in vitro*.

In the current study, the autophagy was induced from 4 to 8 h when ADSCs were grown on TCPS. On CS substrates, ADSCs revealed a greater autophagy response at each same period, and in particular, the autophagy response was prolonged till 24 h (Fig. 1A). After 48 h, the autophagy in CS-derived ADSC spheroids returned to nearly the basal level (Fig. 1A). Therefore, it seemed that the CS substrate did not constitutively stimulate the autophagy by itself but rather enhanced the autophagy response. Before H_2_O_2_ treatment, LC3-II protein expression was not increased in ADSCs grown on CS substrates for 72 h (Fig. 3B). After H_2_O_2_ treatment, the LC3-II protein expression was higher in ADSCs grown on CS substrates than that on TCPS. This result also supported our hypothesis that CS substrates enhanced the autophagy response.

**FIG. 4.**
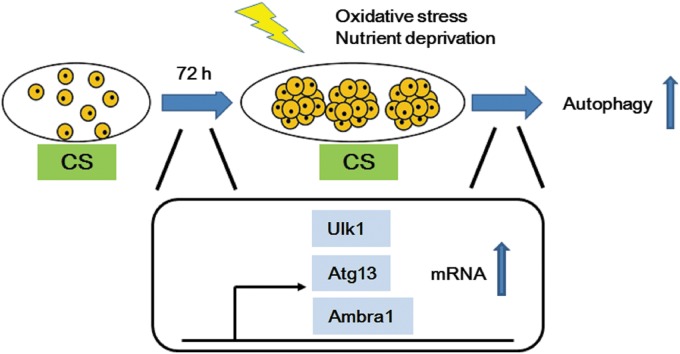
Possible mechanisms for the enhanced autophagy in ADSCs grown on CS substrates. CS substrates may upregulate *Ulk1*, *Atg13*, and *Ambra1* mRNA expression. These proteins are inactivated in basal conditions, while upon H_2_O_2_ stimulation, the autophagy-initiating complex may be activated to enhance the autophagy of ADSCs to protect them against oxidative stress.

According to the literature, Ulk1, Atg13, and Ambra1 were kept in an inactive state by mammalian target of rapamycin complex 1 (mTORC1) phosphorylation before stimulation.^3,4^ Upon autophagy induction, mTORC1 is inhibited and Ulk1, Atg13, and Ambra1 are dephosphorylated and initiate the formation of autophagosomes.^3,4^ In this study, the mRNA expression of components in autophagy-initiating complex increased before H_2_O_2_ treatment in CS-derived ADSC spheroids (Fig. 3C). Even after H_2_O_2_ treatment, the mRNA expression of these genes was higher in ADSCs grown on CS substrates than that on TCPS. Based on the above findings, a possible mechanism is depicted (Fig. 4), where higher mRNA expressions of *Ulk1*, *Atg13*, and *Ambra1* genes may provide more inactivated autophagy-initiating complex in CS-derived ADSC spheroids, which in turn enhances the autophagy activity upon stimulation (Fig. 4).

The sphere formation of ADSCs on CS substrates was impaired by EGTA treatment (Fig. 2A), as observed in our previous report.^24^ Here we demonstrated that EGTA treatment also inhibited autophagy (Fig. 2B). Autophagy was induced from 2 h (Fig. 2A), but at this time cells had not aggregated. In another study, inhibition of basal autophagy in glioblastoma stem cells reduced the number of spheres.^25^ Furthermore, we used the low-adherent bacterial Petri dish (Petri dish) to examine the correlation between autophagy and spheroid formation. The spheroid formation of ADSCs was observed on Petri dish (Supplementary Data and Supplementary Fig. S1A). The LC3-II level of ADSCs on Petri dish was upregulated 2.5-folds than that in TCPS (Supplementary Fig. S1B). However, the LC3-II level of ADSCs on CS increased fourfolds than that in TCPS (Fig. 2B), and the size of ADSC spheroids on CS was more than that on Perti dish.^26^ These findings indicated that the level of autophagy is associated with the efficiency of spheroid formation.

Mammary epithelial cells grown on poly(2-hydroxyethyl methacrylate) (poly-HEMA)-coated plates detached and aggregated because of extracellular matrix deprivation.^27^ In 3D epithelial cultures, autophagy was induced to promote epithelial cell survival, but how the loss of integrin engagement may induce autophagy remains unclear.^27,28^ Activation of AMP-activated protein kinase (AMPK) in detached cells was a possible pathway.^27^ LKB1 activation or increase in Ca^2+^ could activate AMPK and subsequently inhibit mTORC1 to induce autophagy.^7^ Our previous study showed an upregulation of calcium-associated genes in CS-derived MSC spheroids.^29^ CS bound more calcium than TCPS.^24^ Many reports demonstrated that 1,2-bis(o-aminophenoxy)ethane-*N*,*N*,*N*′,*N*′–tetraaceticacid (BAPTA-AM), a Ca^2+^ chelator, blocked autophagy.^30^ In the current study, EGTA also decreased autophagy in a dose-dependent manner (Fig. 2B). These results suggested that Ca^2+^ may play a positive role in the autophagy induction of substrate-derived spheroids.

In conclusion, we found a new method to enhance the stem cell autophagy by 3D spheroid formation on CS substrates. The induced autophagy may equip MSCs with better survival and therapeutic efficacy once they are transplanted.

## Supplementary Material

Supplemental data

## Abbreviations Used

3D: three-dimensional
ADSCs: adipose-derived stem cells
Ambra1: autophagy/beclin-1 regulator 1
AMPK: AMP-activated protein kinase
Atg13: autophagy-related protein 13
CS: chitosan
EGTA: ethylene glycol tetraacetic acid
FBS: fetal bovine serum
LC3: microtubule-associated protein 1 light chain 3
MSCs: mesenchymal stem cells
mTORC1: mammalian target of rapamycin complex 1
TCPS: tissue culture polystyrene
Ulk1: UNC51-like kinase 1
