# Supplementary material for: Enhanced Autophagy of Adipose-Derived Stem Cells Grown on Chitosan Substrates
Source: Biores Open Access. 2015 Jan 1;4(1):89–96. doi: 10.1089/biores.2014.0032 (PMC4497627; doi:10.1089/biores.2014.0032)
Supplement: Supplemental data [file Supp_Data.pdf]

Supplementary Data:

The Autophagy Level of Adipose-Derived Stem Cell Spheroids on Petri Dish

Methods

Adipose-derived stem cells (ADSCs) were seeded on tissue culture polystyrene (TCPS) or Petri dish at a density of 2.5×10^5 cells per well in a 6-well culture plate and were harvested at 8 h. The morphology of ADSC spheroids was observed by an inverted microscope (Leica, DMIRB) and the level of LC3-II was analyzed by Western blots.

Results

The formation of ADSC spheroids was observed on Petri dish, and the LC3-II level of ADSCs on Petri dish was upregulated in 2.5-fold versus TCPS.

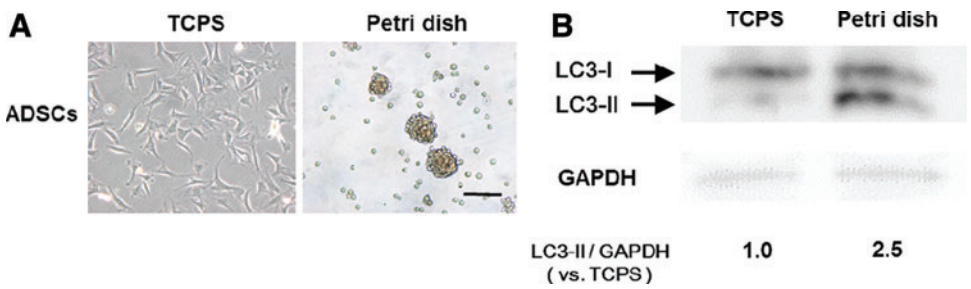

SUPPLEMENTARY FIG. S1. **(A)** ADSCs were seeded on TCPS or Petri dish for 8 h. The morphologies of ADSCs were observed by a microscope. Scale bar = 100 μm. **(B)** Cell lysates were collected for Western blot analysis with anti-LC3 for LC3-II. GAPDH was used as an internal control. Band intensities were quantified and normalized to GAPDH. ADSCs, adipose-derived stem cells; TCPS, tissue culture polystyrene.
